# Establishing the Role of Liver Fatty Acid-Binding Protein in Post-Golgi Very-Low-Density Lipoprotein Trafficking Using a Novel Fluorescence-Based Assay

**DOI:** 10.3390/ijms26062399

**Published:** 2025-03-07

**Authors:** Kayli Winterfeldt, Fahim Rejanur Tasin, Shadab A. Siddiqi

**Affiliations:** Division of Metabolic & Cardiovascular Sciences, Burnett School of Biomedical Sciences, College of Medicine, University of Central Florida, 6900 Lake Nona Blvd., Room# 349, Orlando, FL 32827, USA; kayli.winterfeldt@ucf.edu (K.W.); fahimrejanur.tasin@ucf.edu (F.R.T.)

**Keywords:** very-low-density lipoprotein (VLDL), apolipoprotein B100, post-Golgi VLDL transport vesicle (PG-VTV), Golgi, liver fatty acid-binding protein (LFABP)

## Abstract

The liver plays a crucial role in maintaining lipid homeostasis by converting toxic free fatty acids into VLDL, which the body uses for energy. Even minor changes in VLDL formation and secretion can result in serious health conditions such as atherosclerosis and non-alcoholic fatty liver disease. Despite the importance of VLDL, the proteins and signaling pathways involved in its regulation remain largely unknown. This study aims to develop a novel methodology to study intracellular VLDL transport events and explore the role of liver fatty acid-binding protein (LFABP) in VLDL transport and secretion. Current methods to study VLDL are often tedious, time-consuming, and expensive, underscoring the need for an alternative approach. We designed a new immunofluorescence-based assay to track the formation and secretion of VLDL in cells over time using fluorescently tagged TopFluor oleic acid. Confocal microscopy confirmed that TopFluor oleic acid enters hepatocytes and colocalizes with the ER, Golgi, and plasma membrane. Additionally, the collection of cell culture media revealed that TopFluor was incorporated into VLDL particles, as confirmed by fluorescence readings and ApoB100 immunoblots. This novel assay provides a valuable tool for further research into the mechanisms of VLDL regulation and the development of potential therapeutic targets for related diseases. Utilizing this assay, we identified LFABP as a key regulatory protein in post-Golgi VLDL trafficking. Our data suggest that LFABP plays a crucial role in this process, and its functional impairment leads to reduced VLDL secretion.

## 1. Introduction

The liver plays a critical role in numerous physiological processes in the human body, with one of its primary functions being the maintenance of lipid homeostasis [1]. Lipid homeostasis is crucial for overall health, starting with the removal of free fatty acids (FFAs) from the bloodstream, as high concentrations of FFAs can be harmful to cells. These FFAs are taken up by hepatocytes, where they are converted into triglycerides and subsequently incorporated into very-low-density lipoprotein (VLDL). VLDL is then released into the bloodstream by the liver to provide energy-rich triglycerides to peripheral tissues. It is imperative to note that even minor fluctuations in VLDL production and secretion rates can have significant health implications [2,3,4,5,6,7]. As VLDL travels through the bloodstream and is utilized for energy, remnants of these particles can contribute to the formation of atherogenic plaques in major arteries, which is the primary underlying cause of cardiovascular disease [3,4]. However, when VLDL secretion is impaired, triglyceride-rich lipid droplets can accumulate in the liver, leading to the development of non-alcoholic fatty liver disease (NAFLD) [5,6,7]. Therefore, the tightly regulated process of VLDL formation and secretion is essential for maintaining lipid homeostasis and reducing the risk of serious metabolic disorders. The liver employs three primary pathways to obtain FFAs: first, through uptake from the bloodstream; second, from chylomicron remnants; and third, through de novo lipogenesis (DNL) [2,8,9]. Once inside hepatocytes, FFAs are utilized to synthesize triglycerides in the endoplasmic reticulum (ER), which are then available for the synthesis of new VLDL particles.

The biogenesis of VLDL initiates with the translation and translocation of ApoB100 into the ER. Upon entering the ER, ApoB100 interacts with microsomal triglyceride transferase protein (MTP) to facilitate lipid transfer, resulting in partial lipidation and the formation of the primordial VLDL particle [10,11,12]. This nascent VLDL particle continues to acquire triglycerides as it interacts with ER triglyceride stores. Subsequently, the VLDL particle exits the ER and proceeds to the cis-Golgi for further maturation, a process facilitated by a specialized transport vesicle known as the VLDL transport vesicle (VTV) [11,13]. While the VTV shares some common components with COPII vesicles involved in ER-to-Golgi transport, it possesses unique characteristics that enable it to accommodate larger cargo. It has been demonstrated that proteins such as cideB, SVIP, and reticulon 3 contribute to the formation of this enlarged transport vesicle [14,15,16,17]. The VTV serves multiple functions in VLDL synthesis and trafficking, concentrating both ApoB100 and COPII proteins, protecting the VLDL particle from degradation, and facilitating VLDL delivery to the Golgi for further maturation. In the Golgi apparatus, the VLDL particle undergoes additional modifications, including the glycosylation of ApoB100, particle phosphorylation, the acquisition of additional Apo proteins, and further lipidation [11,12,18,19,20,21,22,23]. Once the mature VLDL particle is ready for secretion, a distinct transport vesicle known as the post-Golgi VLDL transport vesicle (PG-VTV) is formed [24,25]. However, the mechanisms involved in post-Golgi VLDL trafficking remain poorly understood.

Current methods for studying VLDL trafficking are known to be laborious, time-consuming, and costly. Often, these approaches involve expensive animal models or rely on radioactivity, which carries inherent risks and drawbacks, thus limiting the availability of such research [13,25]. While TopFluor-labeled lipids have been previously utilized in various lipid-based studies, including metabolic research, membrane dynamics, and lipid diffusion, their application in VLDL studies has been limited [26,27,28].

Previous research has demonstrated that fluorescently labeled lipids can still undergo metabolism to produce both polar and nonpolar lipid products [29]. Moreover, compared to other fluorescent lipid labels, TopFluor has been shown to have a lesser impact on the biochemical properties of lipids [28]. Given the significance of understanding VLDL trafficking in physiological processes, enhancing the accessibility of research in this area is imperative. To address this need, a new immunofluorescent-based assay was developed utilizing TopFluor-labeled oleic acid. This innovative approach serves as a valuable tool for advancing our comprehension of VLDL trafficking. Through the application of this assay, LFABP was identified as a key regulatory protein in post-Golgi VLDL trafficking.

Our previous in vitro data indicate that the cleavage of liver fatty acid-binding protein (LFABP) by cathepsin B reduces VLDL secretion [30]. However, the mechanism by which LFABP affects VLDL secretion remains to be elucidated. Interestingly, mice lacking functional LFABP genes showed only minor differences compared to wild-type controls when maintained on a standard chow diet containing 4% fat. However, following a 48 h fasting period, these mice exhibited reduced VLDL secretion [31]. LFABP has been implicated in various cellular functions. It has been proposed to contribute to cell division [32], act as a cytosolic reservoir for high levels of unesterified fatty acids [33], and interact with nuclear hormone receptors, potentially facilitating the delivery of specific ligands [34]. These findings underscore the multifaceted nature of LFABP and its significance in diverse cellular processes. In this study, we aimed to explore the mechanism by which LFABP reduces VLDL secretion. Our data suggest that LFABP plays a crucial role in post-Golgi VLDL trafficking, and its functional impairment leads to reduced VLDL secretion.

## 2. Results

### 2.1. Measuring VLDL Secretion Through Fluorescence and ApoB100 Expression

**Figure 1:**
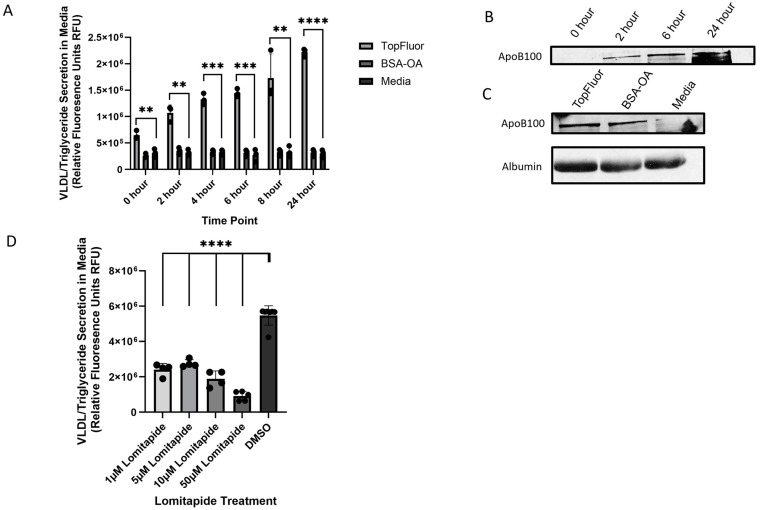
HepG2 cells were treated with 0.1 mM TopFluor OA complexed to BSA-OA in 5% FBS media, 0.1 mM BSA-OA in 5% FBS media, or no OA 5% FBS media. Cells were treated for 1 h before treatment media were removed and cells were washed with PBS, and OA-free 5% FBS media were added back to the cells and incubated for the desired timepoints. Cell culture media were collected and fluorescence was analyzed and then used as a protein sample for immunoblots to detect ApoB100. N ≥ 3. (**A**) Fluorescence analysis over time of cell culture media following treatment with TopFluor OA, BSA-OA, and 5% low-serum cell culture media. For analysis, unpaired *t* tests were run through GraphPad PRISM, and the results were determined to be significant; 0 h ** *p* value 0.0063, 2 h ** *p* value 0.0012, 4 h *** *p* value 0.0001, 6 h *** *p* value 0.0001, 8 h ** *p* value 0.0067, and 24 h **** *p* value < 0.0001. (**B**) Immunoblotting of ApoB100 from collected cell culture media following treatment with TopFluor-labeled OA. Equal volumes of the collected cell culture media from indicated timepoints were used to prepare protein samples to monitor VLDL secretion through the abundance of ApoB100. Samples were run in a 5-well gel due to sample volume. (**C**) Immunoblotting of ApoB100 and Albumin from each treatment group at 8 h post-treatment; no significant difference was observed between TopFluor OA- and BSA-OA-treated cells. (**D**) HepG2 cells were treated with varying concentrations of Lomitapide prior to TopFluor treatment. Lomitapide is a known MTP inhibitor and has been shown to effectively decrease VLDL secretion. All concentrations tested showed a significant decrease determined through an unpaired *t* test in secreted fluorescence associated with VLDL secretion with a *p* value < 0.0001.

HepG2 cells were subjected to various treatments: 0.1 mM TopFluor-tagged OA complexed with BSA-OA, 0.1 mM BSA- OA alone, or low-serum (5% FBS) cell culture media. The concentration of oleic acid remained consistent across all the OA-containing treatment groups. It was anticipated that the groups treated with fatty acids would exhibit similar levels of VLDL secretion over time. With the inclusion of TopFluor in the VLDL formation process, it was presumed that these particles would retain the fluorescent label upon secretion, allowing for the measurement of VLDL secretion through fluorescence analysis. Furthermore, following the initial pulse of OA and the removal of the treatment media, the secretion of VLDL would be the only source of fluorescent accumulation in the media. Upon collecting the cell culture media following treatment, a notable increase in fluorescent intensity over time was observed in the TopFluor-treated group compared to both the BSA-OA treatment and the low-serum media treatment groups. From 0 to 24 h post-treatment, the TopFluor-treated cells exhibited a 2.5–3.5-fold increase in fluorescence intensity in the collected cell culture media, indicating the secretion of the fluorescently tagged VLDL-triacylglycerol (TAG) (Figure 1A). The most substantial difference in fluorescent intensity was noted at 24 h post-treatment, with an average approximate 6.8-fold increase in fluorescent intensity observed in the collected media when comparing treatments. Conversely, no significant change in fluorescence was detected over time in the BSA-OA or media treatment groups, which served as controls for this experiment.

To verify that this signal is associated with VLDL, we recollected the cell culture media following fluorescence analysis and subjected it to immunoblotting for ApoB100, which is commonly used to quantify VLDL secretion. Given that ApoB100 serves as the primary structural protein of VLDL, it is anticipated that, in addition to the rise in TAG secretion over time, there would be a corresponding increase in the ApoB levels measured through immunoblotting. Upon examination of these blots, we observed a progressive elevation in ApoB levels across later timepoints. Specifically, from 0 to 24 h, there was an average 10-fold increase in the ApoB100 protein concentration, as quantified through NIH ImageJ 1.54d analysis (Figure 1B). Comparing the levels of ApoB100 secreted among treatment groups revealed that at 8 h post-treatment, cells treated with TopFluor exhibited a similar VLDL secretion profile to those treated with BSA-OA, whereas the media-treated group exhibited the lowest secretion levels (Figure 1C). This marked elevation in the ApoB100 protein concentration, alongside the increase in the fluorescence signal secreted into the media post-treatment, lends support to our hypothesis that the TopFluor tag is effectively integrated into VLDL particles. Consequently, this fluorescence signal can be employed as a tool for measuring VLDL secretion.

As an additional control to verify that this assay can be utilized for VLDL secretion, HepG2 cells were treated with varying doses of Lomitapide, an MTP inhibitor known for effectively inhibiting VLDL formation and secretion [35,36], before treatment with 0.1 mM TopFluor OA. Four concentrations of Lomitapide were evaluated, and a significant decrease in fluorescence at 24 h post-treatment was observed compared to the DMSO control at every concentration. The highest decrease in fluorescence was observed at 50 µM with an average fold decrease of 0.21 (Figure 1D).

### 2.2. Monitoring Intracellular VLDL Trafficking Through Confocal Microscopy

**Figure 2:**
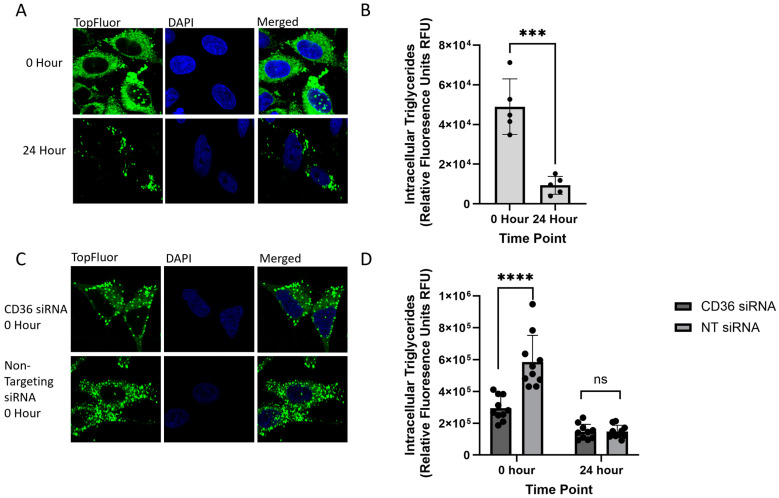
Following treatment with 0.1 mM TopFluor OA complexed with BSA-OA, cells were fixed, stained, and permeabilized on poly-D-lysine-coated coverslips. Cells were imaged through confocal microscopy, and TopFluor signal intensity was recorded at 0 h or 24 h post-treatment. All experiments were performed in triplicate, and ≥5 frames of cells were analyzed. (**A**) Confocal images of TopFluor-treated HepG2 cells 0 h and 24 h post-treatment at 100× magnification. (**B**) Fluorescence intensity of the TopFluor signal at 0 h and 24 h quantified through NIH ImageJ and analyzed through GraphPad PRISM using an unpaired *t* test; *** *p* < 0.0003. (**C**) Confocal images of HepG2 cells treated with either 100 nM siRNA targeting CD36 or non-targeting as a control at 100× magnification. (**D**) Quantification and analysis of fluorescent intensity of the TopFluor signal following siRNA treatment were analyzed through GraphPad PRISM using an unpaired *t* test; **** *p* < 0.0001; ns (*p* value 0.9463).

To further establish this assay as a novel tool for VLDL trafficking research, TopFluor oleic acid was utilized to visualize intracellular VLDL trafficking. TopFluor oleic acid successfully entered hepatocytes and colocalized with organelles involved in VLDL biogenesis and secretion, namely the ER, Golgi, and plasma membrane. As an additional control to verify that TopFluor OA is incorporated into VLDL, colocalization with ApoB100 was also analyzed. Following TopFluor oleic acid treatment, cells were fixed and visualized through confocal microscopy at 0 h, 4 h, and 24 h post-treatment to capture the early and late stages of VLDL trafficking. At 0 h post-treatment, we observed a significant increase in the signal within hepatocytes from the TopFluor oleic acid (Figure 2A,B). As an additional control for this assay, HepG2 cells were treated with 100 nM siRNA to knockdown CD36, a transporter that has been previously implicated in the uptake of FFAs into hepatocytes for VLDL trafficking. With the targeted knockdown leading to a 9% reduction in protein expression, as identified through immunoblots, there was still a significant decrease identified through the statistical analysis in the intracellular TopFluor expression at 0 h, with an average fold decrease of 0.50 (Figure 2C,D).

**Figure 3:**
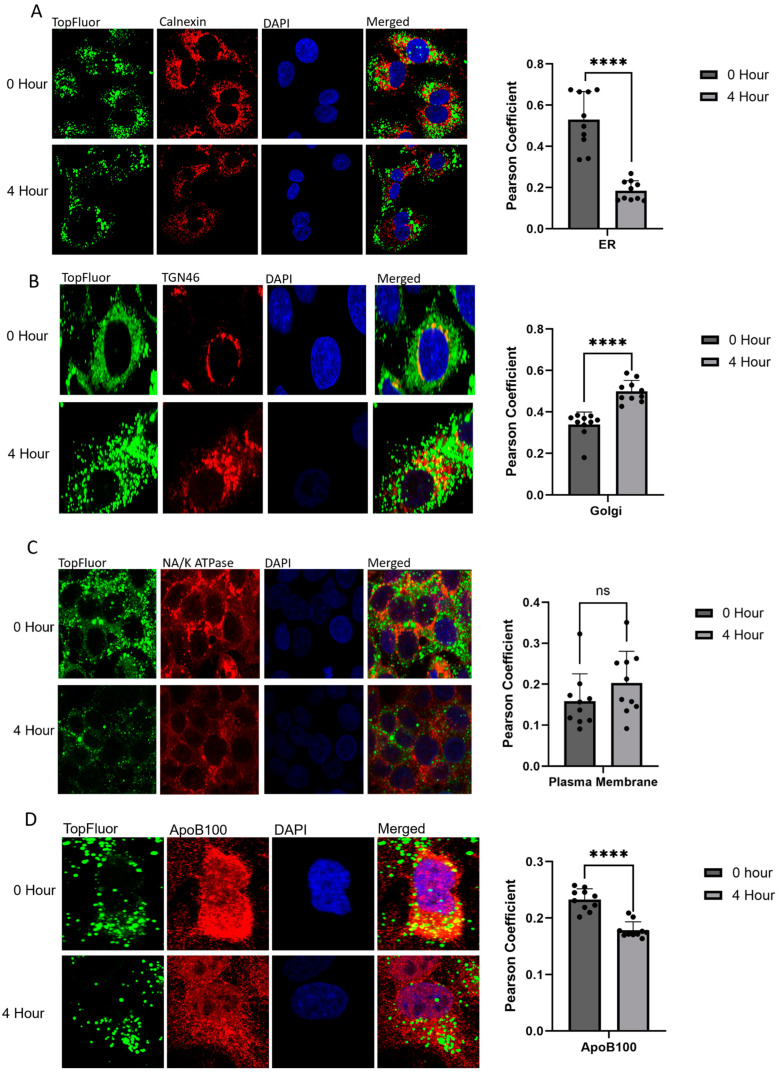
Following treatment with 0.1 mM TopFluor OA complexed with BSA-OA, cells were fixed, stained, and permeabilized on poly-D-lysine-coated coverslips at 0 h and 4 h post-treatment to highlight early stages vs. late stages of VLDL trafficking. Cells were stained for ER, Golgi, plasma membrane, and ApoB100. Colocalization was determined through Pearson’s coefficient calculated through NIH ImageJ. All experiments were performed in triplicate, and ≥10 frames of cells were analyzed. (**A**) Confocal images of HepG2 cells following calnexin staining at 100× magnification. Pearson’s coefficient was calculated through NIH ImageJ, and statistical significance was determined through GraphPad PRISM using unpaired *t* tests; **** *p* < 0.0001. (**B**) Confocal images of HepG2 cells following TGN46 staining at 100× magnification. Pearson’s coefficient was calculated through NIH ImageJ, and statistical significance was determined through GraphPad PRISM using unpaired *t* tests; **** *p* < 0.0001. (**C**) Confocal images of HepG2 cells following Na/K ATPase staining at 100× magnification. Pearson’s coefficient was calculated through NIH ImageJ, and statistical significance was determined through GraphPad PRISM; ns (*p* value 0.4379). (**D**) Confocal images of HepG2 cells following staining for ApoB100 at 100× magnification. Pearson’s coefficient was calculated through NIH ImageJ, and statistical significance was determined through GraphPad PRISM using an unpaired *t* test; **** *p* < 0.0001.

In the early stages of VLDL formation, FFAs are brought into the cell and trafficked to the ER to form triglycerides, which are incorporated into VLDL particles. At this timepoint, there was widespread cytosolic localization, with colocalization observed with the ER (calnexin staining), Golgi (TGN46 staining), plasma membrane (Na/K ATPase staining), and ApoB100 (Figure 3). Colocalization with the ER, plasma membrane, and ApoB100 aligns with the known processes in FFA processing and early VLDL formation. However, colocalization with Golgi at the 0 h timepoint is particularly interesting and may support the hypothesis that additional lipids are added to the VLDL particle within the Golgi during later stages of VLDL trafficking, a topic still under debate [37,38,39,40,41,42,43]. It is also possible that, given the duration of the treatment, there are TopFluor-tagged VLDL particles already in the later stages of their trafficking. At 4 h post-treatment, there is a noticeable decrease in the levels of intracellular fluorescence associated with TopFluor, suggesting that the tagged FFAs have been secreted from the cell in the form of VLDL. Concurrently, there is a reduction in the levels of colocalization observed in both the ER and plasma membrane, coupled with an increase in colocalization with Golgi (Figure 3A–C). This shift is anticipated as a higher population of cells progress to the later stages of VLDL trafficking. At 4 h post-treatment, most of the FFAs are expected to have transported from the apical membrane to the ER and incorporated into VLDL, exited the ER in VTVs, and traveled to the Golgi. At later timepoints, there is a significant decrease in the intracellular fluorescence signal associated with TopFluor, which corresponds to the increased secretion of VLDL into the media. At 24 h post-treatment, there is, on average, a 0.19-fold decrease in fluorescence intensity compared to the 0 h timepoint (Figure 2A,B). Additionally, the localization of the TopFluor signal shifts from a widespread cytosolic pattern to distinct vesicles and lipid droplets (Figure 3), indicating the progression of VLDL trafficking and secretion. With ApoB100 being the main structural protein for VLDL, it is also expected that TopFluor should colocalize with ApoB100, which is seen at both 0 and 4 h post-treatment. It was noted that there is a statistically significant decrease in ApoB100 colocalization from 0 to 4 h post-treatment. It is known that ApoB100 is degraded if not lipidated upon entering the ER. At 0 h post-treatment, there are high levels of circulating lipids for ApoB100’s proper lipidation. At 4 h post-treatment, and with no new lipids entering the cell, ApoB100 is degraded, which can explain the decrease in signal intensity seen and the decrease in colocalization. At this timepoint, VLDL is also secreted into the cell culture media, as observed through the media analysis. ApoB100’s degradation as well as the loss of formed VLDL particles through secretion explain the decrease in colocalization (Figure 3D).

### 2.3. Identifying Changes in the Rates of VLDL Secretion

**Figure 4:**
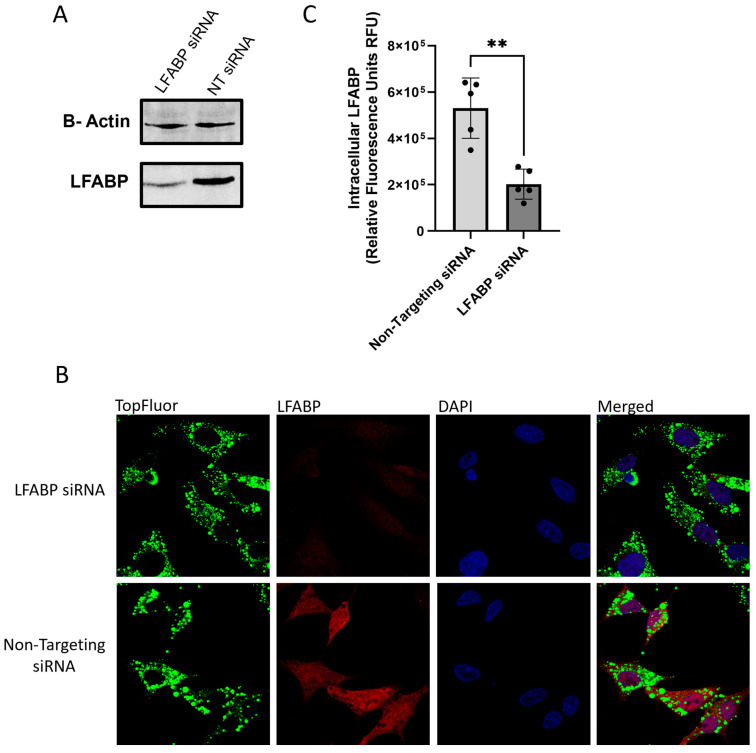
HepG2 cells were treated with lipofectamine RNAimax and 100 nm of siRNA and allowed to incubate for 48 h before treatment with 0.1 mM TopFluor OA complexed with BSA-OA in 5% FBS cell culture media. To verify LFABP knockdown, cells were scraped and collected to prepare protein samples for immunoblots. Furthermore, the LFABP fluorescence signal was determined through confocal microscopy as an additional method of measuring knockdown. All experiments were performed in triplicate, and ≥5 frames of cells were analyzed. (**A**) LFABP and B-actin immunoblot from collected cell lysate following treatment with LFABP siRNA or non-targeting siRNA. (**B**) Confocal microscopy of siRNA-treated HepG2 cells at 100× magnification 4 h post-TopFluor oleic acid treatment. (**C**) Fluorescence analysis of confocal images quantified through NIH ImageJ and statistical significance was determined between LFABP and non-targeting siRNA through GraphPad PRISM using unpaired *t* test; ** *p* < 0.001.

To test the validity of this assay in measuring changes in VLDL trafficking and secretion rates, an siRNA knockdown targeting LFABP was performed. Cells were treated with 100 nM of siRNA and incubated for 48 h before treatment with 0.1 mM TopFluor oleic acid. Knockdown efficiency was assessed through immunoblotting (Figure 4A) and confocal microscopy with TopFluor treatment (Figure 4B,C). Both methods revealed a significant reduction in the LFABP signal. Immunoblotting showed a 0.46-fold decrease in cellular LFABP expression, quantified using NIH ImageJ analysis. Confocal microscopy analysis indicated a 0.38-fold decrease in fluorescence compared to the non-targeting siRNA control. Interestingly, confocal images at 4 h post-treatment revealed distinct patterns between the LFABP and TopFluor signals when comparing the LFABP siRNA treatment to the non-targeting siRNA control. In the control images, distinct rings of LFABP were observed surrounding the TopFluor-labeled VLDL vesicles, highlighting the impact of LFABP knockdown on VLDL trafficking.

**Figure 5:**
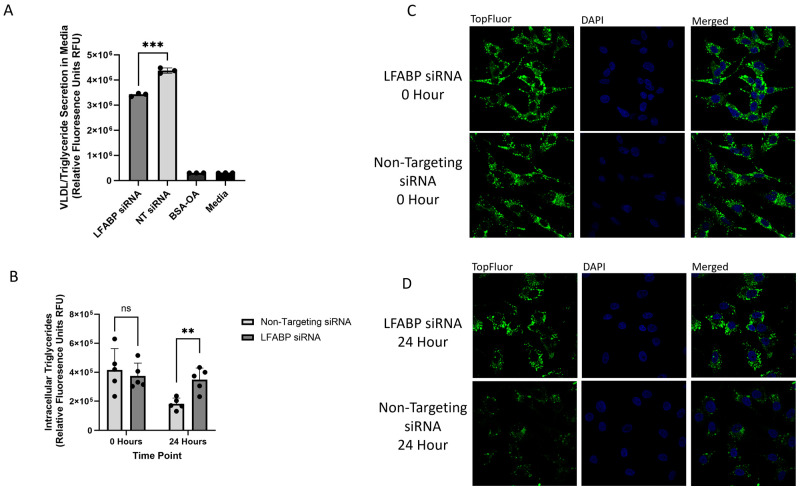
HepG2 cells were treated with lipofectamine RNAimax and 100 nm of siRNA and allowed to incubate for 48 h before treatment with 0.1 mM TopFluor OA complexed with BSA-OA in 5% FBS cell culture media. Cells were incubated with TopFluor media for 1 h prior to cell washing and the replacement of media for incubation of timepoints at 0 and 24 h. Following media collection, cells were fixed, stained, and permeabilized for confocal microscopy. All experiments were performed in triplicate, and ≥5 frames of cells were analyzed. (**A**) Fluorescence analysis of collected cell culture media *** *p* value 0.0002. (**B**) Fluorescence analysis of confocal images quantified through NIH ImageJ with statistical significance determined through GraphPad PRISM using unpaired *t* tests to compare LFABP and non-targeting siRNA at 0 and 24 h timepoints with an ns (*p* value 0.6014) for the 0 h timepoint and a ** *p* value 0.0032 for the 24 h timepoint. (**C**) Confocal microscopy of HepG2 cells at 100× magnification 0 h post-TopFluor treatment following siRNA knockdown (**D**) Confocal microscopy of HepG2 cells at 100× magnification 24 h post-TopFluor treatment following siRNA knockdown.

Following LFABP knockdown verification, the TopFluor assay was performed, and cell culture media were collected 24 h post-treatment. Fluorescence analysis revealed a decrease in secreted fluorescence in the LFABP knockdown cells, showing a 0.79-fold decrease compared to the non-targeting siRNA control cells (Figure 5A). Confocal microscopy analysis of the siRNA-treated cells showed no significant difference in fluorescence intensity at the 0 h timepoint (Figure 5B,C). However, at 24 h, there was a significant increase in the TopFluor signal within the LFABP siRNA-treated cells, indicating an accumulation of VLDL particles due to impaired secretion (Figure 5B,D).

**Figure 6:**
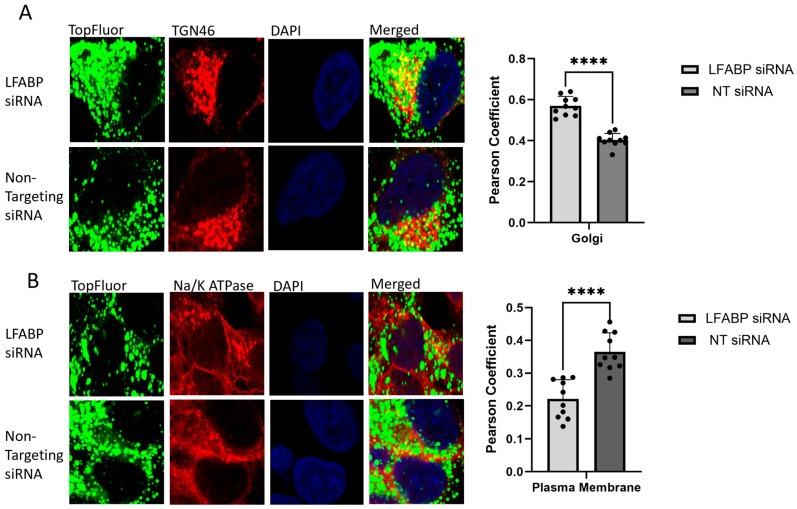
HepG2 cells were treated with lipofectamine RNAimax and 100 nm of siRNA and allowed to incubate for 48 h before treatment with 0.1 mM TopFluor OA complexed with BSA-OA in 5% FBS cell culture media. Cells were incubated with TopFluor media for 1 h prior to cell washing and replacement of media for incubation of timepoints at 4 h. Cells were fixed, stained, and permeabilized for confocal microscopy, and colocalization was determined through calculation of Pearson’s coefficient through NIH ImageJ. All experiments were performed in triplicate, and ≥10 frames of cells were analyzed. (**A**) Confocal microscopy at 100× magnification 4 h post-TopFluor treatment following siRNA knockdown for colocalization of TopFluor and the Golgi through TGN46 staining. Colocalization was quantified through NIH ImageJ analysis, and statistical significance between LFABP and non-targeting siRNA treatment groups was determined through GraphPad PRISM using unpaired *t* test; **** *p* < 0.0001. (**B**) Colocalization of TopFluor and the plasma membrane through Na/K ATPase staining at 100× magnification. Colocalization was quantified through NIH ImageJ analysis, and statistical significance between LFABP and non-targeting siRNA treatment groups was determined through GraphPad PRISM using unpaired *t* test; **** *p* < 0.0001.

To determine the specific stage of VLDL trafficking regulated by LFABP, HepG2 cells were treated with siRNA targeting LFABP and then exposed to 0.1 mM TopFluor oleic acid. At 4 h post-treatment, the cells were fixed and stained to visualize the ER, Golgi, and plasma membrane. The results showed no significant change in the colocalization of the TopFluor signal with the ER. However, there was a significant increase in colocalization with the Golgi and a significant decrease in colocalization with the plasma membrane in LFABP siRNA-treated cells (Figure 6). These data, in conjunction with the observed decrease in secreted VLDL and the increase in intracellular TopFluor signal, suggest that LFABP plays a critical regulatory role in VLDL trafficking and secretion. Specifically, it indicates that LFABP may be involved in the post-Golgi trafficking of VLDL particles. In the absence of functional LFABP, VLDL particles appear to accumulate within the Golgi and fail to be properly transported to the plasma membrane for secretion. The significant decrease in secreted fluorescence in the culture media of LFABP knockdown cells (0.79-fold decrease compared to control) supports this hypothesis. Moreover, the confocal microscopy analysis showed no significant difference in fluorescence intensity at the 0 h timepoint between the knockdown and control cells. However, at 24 h, there was a substantial increase in the TopFluor signal within the LFABP siRNA-treated cells, further indicating an accumulation of VLDL particles due to impaired secretion mechanisms. These findings underscore the importance of LFABP in the proper trafficking and secretion of VLDL particles and validate the utility of the TopFluor assay as a powerful tool for studying VLDL biogenesis and trafficking. The assay not only elucidates the role of LFABP but also demonstrates its effectiveness in investigating the intricate processes of lipid metabolism and transport within hepatocyte.

## 3. Discussion

Despite lipid homeostasis playing a critical role in maintaining human health, the precise mechanisms governing the synthesis and secretion of VLDL remain largely elusive. Even slight alterations in VLDL production and secretion from the liver can lead to severe health complications such as NAFLD and atherosclerosis. Given the substantial portion of the population at risk for developing these diseases, it is crucial that we develop a better understanding of this process to identify potential therapeutic targets for the development of treatments and therapies in the future. Considering the paramount importance of lipid homeostasis research, the current methodologies encounter significant limitations, impeding accessibility to this crucial field of study. This study establishes a novel assay that can be utilized to study VLDL secretion safely and effectively through fluorescence-based techniques.

Through the collection of cell culture media, this assay effectively detected the secretion of fluorescently tagged VLDL particles, a finding further confirmed through ApoB100 immunoblots. What sets this assay apart from other techniques is its unique capability to visualize intracellular VLDL formation and trafficking, as demonstrated by confocal microscopy. The TopFluor tagged FAs exhibited colocalization with the ER, Golgi, plasma membrane, and ApoB100, with changes in localization over time mirroring the known trafficking events during VLDL itinerary. Future studies with this assay could utilize additional markers and timepoints to establish a more detailed kinetics analysis of VLDL trafficking or could potentially be optimized for live cell imaging. Furthermore, this assay successfully pinpointed a change in the rate of VLDL secretion following the siRNA-targeted knockdown of LFABP. Beyond its visualization of intracellular VLDL trafficking steps, this assay serves as a tool to identify potential regulatory proteins involved in VLDL trafficking and secretion. Our findings revealed a significant decrease in VLDL secretion, accompanied by intracellular TopFluor signal accumulation and increased colocalization with the Golgi, alongside a decrease in plasma membrane association. This decrease in secreted VLDL implicates LFABP as a potential regulatory protein in VLDL biogenesis. Moreover, the assay’s capacity for intracellular visualization allowed us to discern the likely step at which LFABP exerts its regulatory influence, indicated by increased Golgi colocalization and subsequent decreased accumulation at the plasma membrane.

The FABP family consists of nine distinct proteins, each named after the tissue in which it was initially identified. These proteins are typically found in high cytosolic concentrations and due to their high binding affinity for fatty acids, one of their primary functions is to mitigate cellular toxicity from elevated levels of FFAs by trafficking them to organelles for processing. Beyond this, various FABP proteins have been discovered to play additional roles in brain development, metabolism, bile acid homeostasis, fertilization, cell signaling, and inflammation [44,45,46]. Previous studies have hypothesized that LFABP may play a significant role in VLDL synthesis and secretion, extending beyond its known function of trafficking FFAs to the ER for processing [30]. In Ames dwarf mice, which exhibit increased levels of liver cathepsin B and lower amounts of LFABP, there is a corresponding decrease in the rates of VLDL secretion [30]. When cells were treated with siRNA targeting cathepsin B, several notable changes occurred, including a 74% increase in LFABP levels and a 115% increase in ApoB, accompanied by increased VLDL secretion rates [30]. Additionally, this study found that LFABP can colocalize with Apolipoprotein B, forming a distinct Golgi-like expression pattern following treatment with oleic acid [30]. Furthermore, LFABP has been hypothesized to play a role in generating larger vesicles necessary for intracellular trafficking during the chylomicron maturation process [47]. It has previously been shown that LFABP displayed pre-chylomicron transport vesicle (PCTV) budding activity from the intestinal ER and that the vesicles formed with LFABP were sealed and contained apolipoproteins [47]. However, LFABP was not found to initiate the budding of COPII-dependent vesicles from the same intestinal ER, suggesting that LFABP might be involved in cargo selection and assisting in the formation of these larger, specialized vesicles [47]. Comparing chylomicron and VLDL trafficking reveals that both processes require the formation of large, specialized transport vesicles to facilitate intracellular transport and particle maturation. In VLDL trafficking, two unique vesicles are formed: the VLDL transport vesicle (VTV), which assists in transporting VLDL from the ER to the Golgi, and the post-Golgi VLDL transport vesicle (PGVTV), which aids in trafficking from the Golgi to the plasma membrane. Typical transport vesicles formed by COPII proteins range from 55 to 70 nm in diameter. In contrast, pre-chylomicrons are approximately 250 nm, VTVs are around 110 nm, and PGVTVs can range from 300 to 350 nm in diameter [11,24,48]. This size difference underscores the unique role that LFABP may play in facilitating the formation and trafficking of these larger vesicles essential for lipid transport and metabolism.

Considering the data obtained from the TopFluor assay with the targeted knockdown of LFABP, along with previously hypothesized roles for LFABP, it is plausible that LFABP could act as a regulatory protein in the formation of the larger PGVTV. The TopFluor assay results demonstrated significant changes in VLDL secretion rates and intracellular trafficking patterns upon LFABP knockdown, suggesting that LFABP might influence the maturation and transport of VLDL particles at a specific intracellular stage. However, while these findings provide compelling evidence for LFABP’s involvement, more research is needed to elucidate its precise role in post-Golgi VLDL trafficking and secretion. Future studies should aim to dissect the molecular mechanisms by which LFABP contributes to the formation and function of PGVTVs. This could involve exploring LFABP’s interaction with other proteins and lipids during vesicle budding and trafficking, as well as its impact on the overall efficiency of VLDL secretion. Understanding these mechanisms in greater detail could provide valuable insights into lipid homeostasis and identify potential therapeutic targets for metabolic diseases associated with dysregulated VLDL secretion, such as NAFLD and atherosclerosis.

## 4. Materials and Methods

### 4.1. Reagents

Human hepatoma cells (HepG2) were purchased from American Type Culture Collection (HB-8065, ATCC, Manassas, VA, USA) and were grown in Dulbecco’s modified Eagle’s medium (DMEM) (Corning, Corning, NY, USA). DMEM was supplemented with 10% fetal bovine serum (FBS) and 1% penicillin/streptomycin (Gibco, Grand Island, NY, USA). Cells were grown at 37 °C with 5% CO_2_. To passage cells, the cells were first rinsed with sterile phosphate-buffered saline (PBS) (Gibco). Then, 0.25% Trypsin-EDTA (Gibco) was added to the cells and incubated at 37 °C for approximately 10 min or until the cells lifted. Fresh cell culture media were then supplemented back in, and the cells were replated.

### 4.2. TopFluor Treatment

HepG2 cells were grown on Poly-D-lysine-coated coverslips (Neuvitro, Camas, WA, USA) and allowed to incubate overnight at 37 °C. TopFluor oleic acid (Avanti Polar lipids, Alabaster, AL, USA) was mixed with oleic acid complexed with bovine serum Albumin (OA-BSA) at a ratio of 1:2 and diluted to a final concentration of 0.1 mM total oleic acid in 5% FBS DMEM. The full serum DMEM was removed from the cells, and cells were rinsed with PBS. The TopFluor oleic acid cell culture media were added to the cells and allowed to incubate for 1 h at 37 °C. The TopFluor treatment was removed, cells were washed with PBS, and fresh untreated 5% FBS DMEM was added back to the cells. Cells were incubated for desired timepoints, ranging from 0 to 24 h, cell culture media were collected, and coverslips were fixed and permeabilized for cell staining.

### 4.3. Fluorescence Measurements

At the desired timepoints, the 5% FBS DMEM was collected in microcentrifuge tubes and measured for FITC fluorescence using the Envision 2104 multilabel reader (Waltham, MA, USA). The fluorescence data were collected and recorded for data analysis following the completion of all the timepoints. Data analysis was performed through GraphPad Prism 10.3.1, employing unpaired *t* tests between the selected groups.

### 4.4. Lomitapide Treatment

Lomitapide, purchased from Sigma Aldritch (St. Louis, MO, USA) (SML1385-5MG), was solubilized in DMSO to create a stock. Twenty-four hours prior to the TopFluor treatment, Hepg2 cells were incubated with a desired concentration of Lomitapide in 5% FBS media overnight. On the day of treatment, the cells were washed with PBS, and the TopFluor assay was performed as normal with the addition of Lomitapide in both the pulse and chase media.

### 4.5. Immunoblotting

Following fluorescence measurements, the cell culture media were recollected in microcentrifuge tubes and used to prepare protein samples. Equal volumes of cell culture medium were used for each sample and added to the Laemmli buffer (Bio-Rad, Hercules, CA, USA), mixed, and boiled for 5 min; samples were centrifuged and loaded into an 8% Acrylamide gel and resolved through SDS-PAGE. Proteins were transferred to a nitrocellulose membrane overnight at 50 mAmps (Bio-Rad), and the membranes were blocked and incubated overnight with the primary antibody. Antibodies for ApoB100 (sc-13538) and Albumin (4929) were purchased from Santa Cruz Biotechnology (Dallas, TX, USA) and Cell Signaling (Danvers, MA, USA), respectively. The following day, the membranes were washed and incubated with HRP-conjugated secondary antibody for 1 h at room temperature. For LFABP and CD36 detection, following siRNA treatment, HepG2 cells were scrapped and collected into a microcentrifuge tube, and the cells were pelleted. Cell culture media were removed, and RIPA buffer (Thermo Scientific, Waltham, MA, USA) was added to the cells, and they were incubated on ice for 5 min. Cells were sonicated and centrifuged for 15 min at 13,000× *g*. The protein concentration was determined through the Bradford assay using protein assay dye (Bio-Rad), and absorbance was calculated with the Beckman Coulter DU800 Spectrophotometer (Brea, CA, USA). A total of 50 µg of protein was added to a 15% acrylamide gel and resolved through SDS-PAGE. Proteins were transferred to a nitrocellulose membrane for 1 h at 80 mAmps, and the membranes were blocked and incubated with primary antibody overnight. Antibodies for LFABP (sc-374537) and B-actin (sc-47778) were both purchased from Santa Cruz Biotechnology. The primary antibody for CD36 was purchased from Abcam (Cambridge, UK) (EPR6573). Proteins were detected using ECL (Thermo Scientific) and autoradiography film (PR1MA by MidSci, Fenton, MO, USA). ImageJ 1.54d analysis was performed to quantify the intensity of the protein bands.

### 4.6. Confocal Microscopy

Coverslips were washed with PBS and incubated in 4% paraformaldehyde (PFA) (Thermo Scientific) for 15 min at room temperature. PFA was removed and coverslips were washed with PBS. Cells were permeabilized with 0.1% Triton X-100 (Sigma St. Louis, MO, USA) for 10 min at room temperature. Coverslips were washed with PBS, and the cells were blocked with 1% BSA (Sigma) in phosphate-buffered saline–Tween20 (PBS-T) for 30 min at room temperature. Primary antibodies for calnexin (MA3-027), TGN46 (ab50595), Na/K ATPase (ab283318), and ApoB100 (sc-13538) were obtained from Invitrogen (Waltham, MA, USA), Abcam (Cambridge, UK), and Santa Cruz Biotechnology (Dallas, TX, USA). Coverslips were incubated with primary antibody overnight at 4 °C. Coverslips were washed and incubated with secondary fluorescent antibodies (sc-2780, Santa Cruz Biotechnology) and A11005 (Invitrogen) for 1 h at room temperature. Coverslips were washed for a final time before mounting with Dapi-Fluoromount-G™ Clear Mounting Media (Southern Biotech, Birmingham, AL, USA). The mounted microscope slides were allowed to dry for 24 h before imaging. Images were taken with and processed with Leica TCS SP5II (Wetzlar, Germany) and LAS X office version 1.4.4.26810. The fluorescent intensity was calculated through ImageJ.

### 4.7. siRNA Treatment

HepG2 cells were transfected using lipofectamine siRNA Max (Thermo Fisher, Waltham, MA, USA). A transfection was performed following the manufacturer’s protocol and recommendations. siRNA was purchased from Horizon (Lafayette, CO, USA) (L008678-00-0005, L-010206-00-0005 and D001810-10-05). siRNA was used at a final concentration of 100 nM. Cells were allowed to incubate for 48 h post-transfection before knockdown validation by Western blot, as well as continuing treatment with TopFluor.

### 4.8. Statistical Analysis

Statistical analysis of the data was performed using an unpaired *t* test through GraphPad PRISM version 10 software.

## Data Availability

All data are presented in the manuscript and raw data (such as immunoblots, etc.) will be available upon request.
